# The effect of perceived spousal support on childbirth self-efficacy on pregnant women in turkey

**DOI:** 10.1186/s12884-023-05508-6

**Published:** 2023-03-14

**Authors:** Burcu Kucukkaya, Sukran Basgol

**Affiliations:** 1 Department of Women Health and Diseases Nursing, Nursing Department, Trakya University, Faculty of Health Sciences, Edirne, Turkey; 2 Trakya University Rectorate, 22030 Balkan Campus, Edirne, Turkey; 3 Midwifery Department, Ondokuz Mayis University, Faculty of Health Sciences, Samsun, Turkey

**Keywords:** Pregnant, Spousal support, Childbirth self-efficacy

## Abstract

**Background:**

Spousal support during pregnancy reduces the anxiety and stress of the pregnant women and increases the ability to cope with the problems experienced by the pregnant women. The study aimed to examine the effect of perceived spousal support on childbirth self-efficacy on pregnant women in Turkey.

**Methods:**

This cross-sectional exploratory study was conducted on 524 pregnant women who volunteered to participate in the research by approving the shared online survey and distributed by sharing the online questionnaire created by the researchers on social media (such as Facebook and Instagram) forums or group pages between 20 June and 27 July 2022. Data were collected with an e-questionnaire including The Information Form prepared by examining the literature from the pregnant women who volunteered to participate, the Spouse Support Scale measures the perceived spousal support level and consists of 27 questions, and the Childbirth Self-Efficacy Scale measures women's self-confidence in childbirth and their ability to cope and consists of 32 questions in the study.

**Results:**

The mean age of the pregnant women was 29.99 (5.62) and the mean week of gestation was 25.93 (7.72). It was determined that 11.5% of the pregnant women were related to their spouses, 78.2% were married voluntarily, 86.5% had knowledge about birth, and 74.6% had a planned pregnancy. The Spouse Support Scale (SSS) total score average was 66.06 (19.82), the emotional support sub-dimension mean score of SSS was 22.03 (6.63), the material support and information support sub-dimension mean score was 17.17 (5.12), appreciation support sub-dimension mean score was 19.52 (5.90) and social interest support sub-dimension mean score was 21.98 (10.18), while the total mean score of the Childbirth Self Efficacy Scale (CBSES) was 234.20 (108.14), CBSES's result expectation sub-dimension mean score was 116.98 (54.13), and proficiency expectation sub-dimension mean score was 117.22 (54.07). A statistically significant correlation was found between the total and sub-dimension mean scores of CBSES and the mean scores of the total and sub-dimension SSS in pregnant women (*p* < .001).

**Conclusion:**

It was determined that perceived spousal support has an effect on birth self-efficacy in pregnant women in Turkey. Obstetrics nurses and midwives should support the spousal support and birth self-efficacy of pregnant women during pregnancy follow-up and delivery.

## Background

The birth process is a challenging life event as pregnant women face not only new problems but also various biological, physiological, and psychological changes. However, it is of great importance for many women as the birth process represents a magical moment with their newborn baby [1]. Childbirth self-efficacy is defined as the dynamic cognitive process of an individual's confidence in her ability to cope with a childbirth event [2]. Pregnant women’s focus on birth and their babies is a motivating factor and ensures that the birth process progresses according to the mother’s expectations [3]. Woman’s higher self-efficacy during labor, the development of methods to cope with the pain caused by stress and contractions, and the transformation of these methods into behavior facilitate the birth process and lead to positive emotions in women [4, 5]. With the increase in the level of anxiety in mothers with low birth self-efficacy, the presence of postpartum depression symptoms increases the possibility of experiencing post-traumatic stress disorder in the postpartum period [6]. In line with this result, childbirth self-efficacy has a significant impact on a woman's birth experience, facilitating her ability to cope with the birth process and strengthening her confidence in managing any uncertainty about childbirth [2].

Regarding the literature, childbirth self-efficacy is sensitive to many factors, including socio-demographic factors (e.g., age, education level, gestational week, the experience of pregnancy loss, and the number of births), psychological factors, complex obstetric factors, religious-cultural factors [7, 8]. Pregnancy is a more special process, just as being a woman is important in Islam. The fact that most of the religious belief in Turkey is Islam, and the Turkish culture and traditions according to the religion of Islam makes pregnancy important. Besides, self-efficacy perception is negatively affected by psychological conditions such as the woman's fear of childbirth, lack of spouse and family support, and the stories told about other women's experiences during childbirth. Women with low childbirth self-efficacy may have a limited ability to motivate themselves to adapt to the birth experience [7, 9].

Being one of the factors that affect childbirth self-efficacy and create stress in women, spousal support not only affects the pregnant women and the fetus but also affects the bond and communication between the pregnant women and her husband [10]. The introversion of some pregnant women may cause negative situations such as communication problems between couples, distance from each other, inability to ehossxpress their feelings towards each other, and not being able to receive support from each other. In addition to the pregnancy process and these negative situations, the restriction or prohibition of sexual intercourse may cause pregnant women to feel guilty towards their spouses, to evaluate the pregnancy process negatively, and to cause jealousy between spouses [11, 12].

Husband support is one of the most important factors affecting the social support system. The literature argues that married couples first apply to their spouses when they have stress or encounter a problem and that couples see their spouses as the main source of social support [13]. Researchers advocate that spousal support (received during pregnancy, delivery, and postpartum processes) prevents the isolation and withdrawal that may occur between spouses in stressful times, that a strong bond is established between the spouses with emotional intimacy thanks to this support, and that calming is achieved and increasing negative situations can be prevented by avoiding situations that will create an environment of conflict [7, 14, 15]. Spousal support during pregnancy reduces the anxiety and stress of the pregnant women, and as the support of the spouse increases, the ability to cope with the problems experienced by the pregnant women increases [14]. Research reports that receiving spousal support during pregnancy affects the perception of motherhood positively and that they could cope with the stressful factors experienced during pregnancy more easily [15].

Childbirth self-efficacy and spousal support are considered basic concepts in midwifery and nursing care, as they focus on the importance of reinforcing spousal support and childbirth self-efficacy [16]. Counseling given by nurses during pregnancy and evaluating childbirth self-efficacy and spousal support of pregnant women may positively affect the birth process. It can reinforce maternal-infant bonding and family ties. Therefore, women health or reproductive health nurses should plan interventions and training to increase childbirth self-efficacy and spousal support for pregnant women [17, 18]. Considering that factors related to childbirth self-efficacy and spousal support may differ depending on socio-cultural factors and differences according to regions, it is more accurate to determine factors related to childbirth self-efficacy and spousal support in a specific environment [19–21]. This study aimed to examine the effect of perceived spousal support on childbirth self-efficacy on pregnant women in Turkey. Our study is original in that it examines the effect of spousal support on childbirth self-efficacy and reveals its levels in Turkish society together. In the national and international literature, there is no study examining the effect of spousal support of pregnant women on childbirth self-efficacy, and the study will support the literature with this feature.

## Methods

### Research Design and Participants

This study is a descriptive exploratory study and was conducted using a web-based online survey between June and July 2022 on pregnant women who use social media applications such as Facebook, Instagram, Telegram, or Whatsapp and are members of pregnancy groups and forums. It was calculated that 524 pregnant women were required to be included in the study in order to determine the scale score of the “Spousal Support Scale” score with a standard deviation value of 1.21, a confidence level of 99%, and a margin of error of 1% [22]. Twenty-four pregnant women (4.4%) who did not meet the inclusion criteria of the study (high risk pregnancy = 10; older than 49 years of age = 2) and who completed the questionnaires incompletely (*n* = 12) were excluded from the study. All (*n* = 524; 100.0%) of the sample determined in the study were reached. The study was not stopped until 524 pregnant women were calculated to complete the sample power analysis. The inclusion criteria were (1) over the age of 18, (2) could read and write, (3) had technological equipment such as computers or phones, (4) were members of pregnancy groups via web applications (Facebook, Instagram, Telegram or Whatsapp), and (5) were willing to participate in an online survey. The exclusion criteria were (1) older than 49 years of age, (2) illiterate, (3) not having technological equipment such as computers or phones, (4) pregnant women with high risk pregnancy, and (5) not being willing to participate in an online survey.

### Data Collection

Pregnant women who are members of prenatal or pregnant women groups using social media (Facebook, Instagram, Telegram and Whatsapp) were invited to participate in the study via a shared online survey link. The first page of the shared online questionnaire contains a question evaluating the purpose of the study, information about the study, and the willingness of pregnant women to participate freely in the study.

### Data Collection Instruments

The data were collected online using a sociodemographic and obstetric characteristic questionnaire, the Spousal Support Scale (SSS), and the Childbirth Self-Efficacy Scale (CBSES).

### The first section of the survey involves an assessment of sociodemographic variables

Sociodemographic and obstetrics data included age, marriage age, marriage duration, education level, working position throughout pregnancy, existence of a chronic condition, frequent physical activity, pregnant women smoking, education level of a spouse, employment status of spouse, state of being related to one's spouse, the status of marrying voluntarily, the status of being compatible with spouse [19, 21, 23].

### The second section of the survey: significance values regarding the obstetrics characteristics

The obstetrics characteristics data included gestational week, number of pregnancies, number of living children, the status of planned pregnancy, mood related to pregnancy, the status of going for control during pregnancy, breastfeeding experience, education about breastfeeding, spousal support for housework during pregnancy, support of close people such as relatives and friends in housework during pregnancy [19, 21, 23].

### The third section of the survey is an assessment of spousal support

The Spousal Support Scale (SSS) was developed by Yildirim (2004) to assess the level of perceived spousal support. The SSS consists of 27 items scored on a 3-point Likert-type scale (“1 = Does not describe me at all” to “3 = Describes me well”). The SSS has four subscales: (1) emotional support, (2) instrumental and informational support, (3) appraisal support, and (4) social support. The total score ranges from 27 to 81, with higher scores indicating higher perceived spousal support [24]. The SSS has a Cronbach’s alpha of 0.95 in its original form, the Cronbach’s alpha was found to be 0.99 in this study.

### The fourth section of the survey is an assessment of childbirth self-efficacy

The Turkish validity and reliability study of the Childbirth Self-Efficacy Scale (CBSES), which measures women's self-confidence in childbirth and their ability to cope, was conducted by Ersoy (2011) [25]. The scale has two sub-dimensions, outcome and sufficiency expectation, and consists of 32 questions in total. Answers are given on a Likert-type scale from 1 to 10. The lowest score obtained from the scale is 32, and the highest score is 320. The high scores obtained from the scale indicate that the pregnant women's self-efficacy levels in childbirth are high. Ersoy (2011) found Cronbach's alpha coefficient of the scale to be 0.90 [25]. The CBSES's Cronbach's alpha coefficient was determined to be 0.99, suggesting high reliability.

### Ethics approval and consent to participate

It was abided by the Declaration of Helsinki on research on human subjects and the Universal Declaration on Human Rights throughout the study. The Trakya University Scientific Research Ethics Committee (TUTF-GOBAEK 2022‐317) approved the present study. An electronic informed consent was presented on the first page of the online survey. The participants were electronically informed on the first page of the survey that they were volunteering to participate and that they could withdraw from the survey at any time.

### Data Analyses

The Shapiro-Wilk test was used to evaluate the normal distribution of the data in the study. Descriptive data are shown as mean, standard deviation, median (min-max), percentage and numbers. Differences between non-normally distributed data were examined using Mann-Whitney U (z) and Kruskal-Wallis H tests. In addition, Post hoc power analysis was also performed. The relationship between SSS and CBSES was examined using Spearman correlation analysis. Correlations between factors and subscales were determined using linear regression analysis. Cronbach's alpha was used to show the internal consistency coefficient of the scales. IBM SPSS Statistics for Windows, Version 23.0 (IBM Corp) was used for statistical analysis. Statistical significance was defined as *p* < 0.05.

## Results

### Participants' demographic, pregnancy process, and spouse-related obstetrical variables

Table 1 shows demographic and obstetric characteristics. The pregnant women's mean age was 29.99 (5.62) (range, 18-45 years), their mean marriage year was 7.32 (5.66), and their mean week of gestation was 25.93 (7.72). It was determined that 78.2% of them married voluntarily (Table 1).

**Table 1:** Demographic and obstetrics characteristics of the pregnant women

Valiables	*n* = 524
**Age, years, mean ± SD**	29.99 ± 5.62
**Marriage years, mean ± SD**	7.32 ± 5.66
**Gestational week, mean ± SD**	25.93 ± 7.72
**Number of pregnancies, mean ± SD**	2.00 ± 1.29
**Number of living children, mean ± SD**	0.82 ± 1.05
**Miscarry**	0.16 ± 0.71
**Curettage**	0.04 ± 0.23
**Education, years, n (%)**	
< 9	55 (10.5)
≥ 9	469 (89.5)
**Income status**	
Low	53 (10.1)
Middle	460 (87.8)
High	11 (2.1)
**Working status during pregnancy, n (%)**	
Yes	146 (27.9)
No	336 (64.1)
Maternity leave	42 (8.0)
**Chronic illness, n (%)**	
Yes	37 (7.1)
No	487 (92.9)
**Regular physical activity, (walking or pregnancy exercise), n (%)**	
Yes	266 (50.8)
No	258 (49.2)
**Smoking during pregnancy, n (%)**	
Yes	35 (6.7)
No	489 (93.3)
**Place of residence, n (%)**	
Province	411 (78.4)
District	113 (21.6)
**Family type**	
Nucleus	473 (90.3)
Expend	51 (9.7)
**Education of spouse, years, n (%)**	
< 9	5 (1.0)
≥ 9	519 (99.0)
**Working status of spouse, n (%)**	
Yes	518 (98.9)
No	6 (1.1)
**Status of marrying willingly, n (%)**	
Yes	410 (78.2)
No	114 (21.8)

It was determined that 88.5% of the pregnant women were not related to their spouses, and 70.2% of them were compatible with spouses. Moreover, 64.1% of the participants reported that their spouses support housework during pregnancy, and 12.0% of them reported that their close people, such as relatives and friends, support housework during pregnancy (Table 2).

**Table 2:** Pregnancy process and spouse-related characteristics of the pregnant women

Valiables	*n* = 524
**Status of being compatible with spouse, n (%)**	
Yes	368 (70.2)
No	156 (29.8)
**Status of being related to one's spouse, n (%)**	
Yes	60 (11,5)
No	464 (88,5)
**Status of planned pregnancy, n (%)**	
Yes	391 (74.6)
No	133 (25.4)
**Mood related to pregnancy, n (%)**	
Positive	407 (77.7)
Negative	51 (9.7)
Uncertain	66 (12.6)
**Status of going for control during pregnancy, n (%)**	
Yes	469 (89.5)
No	55 (10.5)
**Breastfeeding experience, n (%)**	
Yes	241 (46.0)
No	283 (54.0)
**Education about breastfeeding, n (%)**	
Yes	455 (86.8)
No	69 (13.2)
**Spousal support for housework during pregnancy, n (%)**	
Yes	336 (64.1)
No	188 (35.9)
**Support of close people such as relatives and friends in housework during pregnancy, n (%)**	
Yes	63 (12.0)
No	461 (88.0)

### Spousal support and childbirth self-efficacy in pregnant women

The average SSS score of the participants was 66.06 (19.82), and the average CBSES score was 234.20 (108.14) (Table 3).

**Table 3:** Spousal support and childbirth self-efficacy in pregnant women

	*Mean ± SD*	*Median* *(Min–Max)*
**SSS**	66.06 ± 19.82	81 (27–81)
Emotional support	22.03 ± 6.63	27 (9–27)
Financial and information support	17.17 ± 5.12	21 (7–21)
Appreciation support	19.52 ± 5.90	24 (8–24)
Social interest support	21.98 ± 10.18	30 (3–30)
**CBSES**	234.20 ± 108.14	320 (32–320)
Outcome expectancy	116.98 ± 54.13	160 (16–160)
Self-efficacy expectancy	117.22 ± 54.07	160 (16–160)

*Abbreviations*: *SSS *Spousal support scale, *CBSES *Childbirth self-efficacy scale

### The correlation between pregnant women's Spousal support and childbirth self-efficacy

The total score of the SSS and the total score of the CBSES were found to be strongly positively correlated (*r* = .925, *p* < .001), and statistically significant strong positive correlations were observed between the average score of the “outcome expectancy” subscale (*r* = .926, *p* < .001), and the average score of the “self-efficacy expectancy” subscale (*r* = .923, *p* < .001) of the CBSES scale (Table 4).

**Table 4:** The relationship between the Spousal support and childbirth self-efficacy in pregnant women

		1	*2*	*3*	*4*	*5*	*6*	*7*	*8*
**CBSES (1)**	*r* *p*	1							
Outcome expectancy (2)	*r* *p*	0.999* **< 0.001**	1						
Self-efficacy expectancy (3)	*r* *p*	0.999* **< 0.001**	0.998* **< 0.001**	1					
**SSS (4)**	*r* *p*	0.925* **< 0.001**	0.926* **< 0.001**	0.923* **< 0.001**	1				
Emotional support (5)	*r* *p*	0.927* **< 0.001**	0.928* **< 0.001**	0.925* **< 0.001**	0.998* **< 0.001**	1			
Financial and information support (6)	*r* *p*	0.921* **< 0.001**	0.922* **< 0.001**	0.919* **< 0.001**	0.998* **< 0.001**	0.993* **< 0.001**	1		
Appreciation support (7)	*r* *p*	0.921* **< 0.001**	0.922* **< 0.001**	0.919* **< 0.001**	0.998* **< 0.001**	0.994* **< 0.001**	0.996* **< 0.001**	1	
Social interest support (8)	*r* *p*	0.998* **< 0.001**	0.998* **< 0.001**	0.997* **< 0.001**	0.921* **< 0.001**	0.923* **< 0.001**	0.918* **< 0.001**	0.917* **< 0.001**	1

### The effect of the SSS and its subscales on CBSES

The effect of SSS and its subscales on CBSES is seen in Table 5. The degree of perceived spousal support is a crucial predictor of childbirth self-efficacy. The disclosure rate with these skills was 93% (*F*_*(1)*_ = 3104.859, *p* < .001) (Table 5).

**Table 5:** Linear regression analysis related to prediction of the SSS by the CBSES Scale and its subscales

Variables	ß	Std. error	β	*t*	*p*
**Fixed**	26.357	0.785		33.588	0.000
**The CBSES total**	0.170	0.003	0.925	55.721	0.000
R = 0.93, R^2^ = 0.86, F = 3104.859, p < 0.001					

*SSS *Spousal support scale, *CBSES *Childbirth self-efficacy scale

Dependent variable: SSS, significance variable for *0.05

## Discussion

This study investigated the effect of perceived spousal support on childbirth self-efficacy.

While the results made a significant contribution to the literature on spousal support and childbirth self-efficacy in pregnant women, they also reported important information about the cultural aspects of Turkish women's perceptions of spousal support during pregnancy.

In this study, the level of spousal support was determined to be high. Regarding studies using this scale, the mean total score was determined as 68.99 (SD 10.8), with high spousal support [26]. Besides, it was found 57.69 (SD 9.21) in the study conducted by Ozbek and Beydag (2019) with high-risk pregnant women, and 81.55 (SD 11.87) in the study conducted by Lee et al. (2019) with pregnant women [27, 28]. In the study of Arisukwu et al. examining the level of spousal support received by pregnant women living in Nigeria, it was reported that 93.2% of pregnant women received spousal support [29]. Accordingly, this study confirms the literature, indicating that pregnant women receive high spousal support. Spousal support during pregnancy is the most important source of social support that accompanies women's sense of respect, security, and self-confidence. Hence, one of the frequently used sub-dimensions of the Multidimensional Scale of Perceived Social Support is spouse/significant others. In a study conducted in Iran to determine the relationship between perceived social support and birth experiences in pregnant women, spouse/significant others was found to be the highest perceived social support dimension by pregnant women [30]. In a systematic review study in which 18 studies were evaluated, it was emphasized that spousal support shaped the pregnancy and birth process positively and increased the satisfaction of pregnant women [31]. In a systematic review and meta-analysis study, the importance of spousal support during pregnancy was emphasized, and it was found to be related to the mental health status and feelings of competence of pregnant women [32]. Pregnant women needed spousal support, which that considered one of the most important factors affecting their mood during pregnancy [33]. On the other hand, pregnancy is very valuable for a woman in Turkish culture, and motherhood has a religious basis in Islam. There are hadiths in the Qur'an regarding the pregnancy process and spousal support. Most of the religious belief in Turkey is Islam, so Turkish culture and traditions according to the religion of Islam [34]. It is thought that these religious and cultural beliefs could also contribute to the highly perceived spousal support of Turkish pregnant women.

In the present study, the level of childbirth self-efficacy was found to be high. The literature has reported that CBSES scores vary from country to country and from culture to culture. For example, it was 244.27 (SD 45.12) in a study conducted with 380 pregnant women in Turkey [19], 212.03 (SD 59.64) in a study conducted with 347 pregnant women in China [35], and 228.7 (SD: 40.9) in a study conducted with 425 pregnant women in Uganda [36]. Based on these data, the result of the current study was found to be higher. In Turkey, antenatal training has been provided free of charge to all pregnant women due to the increasing recent cesarean section rates. Thanks to the training, women receive information about pregnancy, childbirth, and breastfeeding, and also their childbirth self-efficacy levels increase [19]. Although there was no significant difference between the sociodemographic variables of the women and the mean total score of the scale, almost all participants had an education level of 9 years or more. It was observed that pregnant women with high education levels had higher self-management skills, awareness, childbirth self-efficacy, and communication skills [37]. The literature recommends prenatal counseling by midwives/nurses, making prenatal education classes accessible, and providing motivational interviews with pregnant women and their spouses to increase women's childbirth self-efficacy [19, 35, 36]. Midwives/nurses should organize a comprehensive education considering the factors that increase the birth self-efficacy of pregnant women, and one of these variables is spousal support.

This study argues that the perceived spousal support of pregnant women positively affects their childbirth self-efficacy. As spouse support increases in pregnant women, their childbirth self-efficacy also increases. Spouses have a special role in promoting the health of mothers and babies during pregnancy, childbirth, and the postpartum periods. Pregnant women receiving more spousal support have increased positive emotions and exhibit high-quality health behaviors. Besides, they benefit more from antenatal care services, adapt to pregnancy more easily, and develop feelings of self-efficacy [37].

### Limitations of the research

The current study contains a few limitations. The first limitation was that because it was a web-based study, pregnant women who could fill out the online questionnaires had similar socioeconomic levels. In addition, the participants consisted of only pregnant women living in Turkey. Therefore, the findings of the study may not be generalizable because of social differences. The second limitation was since this study is a web-based, only motivated pregnant women will be able to answer the scales, as pregnant women who have internet access and use social media can be reached. Therefore, there may be a potential risk of bias or imprecision. The third limitation was the use of the cross-sectional exploratory design. It is difficult to determine the reasons for the correlation between the variables, as it is carried out within the scope of longitudinal designs

## Conclusions

It was determined that perceived spousal support has an effect on birth self-efficacy in pregnant women in Turkey. Moreover, it was found that SSS and CBSES were closely related to each other. The present study argues that as spouse support increases in pregnant women, childbirth self-efficacy increases.

Childbirth self-efficacy directly affects the pregnancy, birth and postpartum periods of the woman and the general well-being of the family. A healthy family creates a healthy society. Therefore, obstetrics nurses and midwives should not neglect to consider perceived spousal support while taking initiatives to increase the self-efficacy perception of pregnant women. The study findings contribute to the literature, indicating that nurses should get information about perceived spousal support during the evaluation process of pregnant women and underlying the importance of the effect of spousal support on childbirth self-efficacy in pregnant women. Thus, it is recommended to activate prenatal training, cognitive behavioral therapies, mindfulness training, and motivational interviews in which couples participate together.

## Data Availability

The datasets used and/or analyzed during the current study are not publicly available due to the sensitive nature of the interviews. If someone wants to request the data from this study, the corresponding author should be contacted.

## Abbreviations

SSS: Spouse Support Scale
CBSES: Childbirth Self Efficacy Scale
SD: Standard Deviation
